# The F-box protein Bard (CG14317) targets the Smaug RNA-binding protein for destruction during the *Drosophila* maternal-to-zygotic transition

**DOI:** 10.1093/genetics/iyab177

**Published:** 2021-10-20

**Authors:** Wen Xi Cao, Angelo Karaiskakis, Sichun Lin, Stephane Angers, Howard D Lipshitz

**Affiliations:** 1 Department of Molecular Genetics, University of Toronto, Toronto, ON M5G 1M1, Canada; 2 Department of Pharmaceutical Sciences & Donnelly Centre, University of Toronto, Toronto, ON M5S 3E1, Canada

**Keywords:** Smaug, RNA-binding protein, E3 ubiquitin ligase, Skp/Cullin/F-box (SCF) complex, Bard/CG14317

## Abstract

During the maternal-to-zygotic transition (MZT), which encompasses the earliest stages of animal embryogenesis, a subset of maternally supplied gene products is cleared, thus permitting activation of zygotic gene expression. In the *Drosophila melanogaster* embryo, the RNA-binding protein Smaug (SMG) plays an essential role in progression through the MZT by translationally repressing and destabilizing a large number of maternal mRNAs. The SMG protein itself is rapidly cleared at the end of the MZT by a Skp/Cullin/F-box (SCF) E3-ligase complex. Clearance of SMG requires zygotic transcription and is required for an orderly MZT. Here, we show that an F-box protein, which we name Bard (encoded by CG14317), is required for degradation of SMG. Bard is expressed zygotically and physically interacts with SMG at the end of the MZT, coincident with binding of the maternal SCF proteins, SkpA and Cullin1, and with degradation of SMG. shRNA-mediated knock-down of Bard or deletion of the *bard* gene in the early embryo results in stabilization of SMG protein, a phenotype that is rescued by transgenes expressing Bard. Bard thus times the clearance of SMG at the end of the MZT.

## Teaser

Degradation of the Smaug RNA-binding protein is essential for an orderly maternal-to-zygotic transition (MZT) and requires zygotic transcription. Cao *et al*. show that the F-box protein Bard is expressed in a narrow time window at the end of the MZT. Upon Bard expression, an SCF E3 ubiquitin ligase complex binds to Smaug, and Smaug is degraded. Loss of Bard results in failure to degrade Smaug, meaning Bard serves as a zygotic timer for Smaug clearance and an orderly MZT.

## Introduction 

The earliest stages of animal embryogenesis depend on maternally supplied factors, including mRNAs and proteins, that are loaded into the oocyte. During a conserved process known as the oocyte-to-embryo or maternal-to-zygotic transition (MZT), a large proportion of the maternally loaded mRNA and a much smaller proportion of protein species is degraded in a temporally co-ordinated manner, permitting transcriptional activation of the zygotic genome (reviewed in Tadros and Lipshitz 2009; Vastenhouw *et al.* 2019). Prior to zygotic genome activation (ZGA), the regulation of maternal mRNAs and proteins relies on post-transcriptional and post-translational mechanisms implemented by maternally encoded machineries whereas, upon ZGA, newly synthesized factors are added to the mix (reviewed in Vastenhouw *et al.* 2019). For example, degradation, and/or translational repression of maternal mRNAs prior to ZGA is directed largely by maternally encoded RNA-binding proteins while, upon ZGA, microRNAs are synthesized and destabilize additional maternal mRNAs (*e.g.*, *miR-430* in zebrafish and *miR-309* in *Drosophila*). Post-translational modifications that occur during the MZT include phosphorylation and ubiquitylation, which affect protein function and/or stability.

In *Drosophila melanogaster*, the multifunctional RNA-binding protein Smaug (SMG) acts in concert with various cofactors to direct translational repression and/or degradation of a large fraction of the maternally loaded mRNA species (Nelson *et al.* 2004; Semotok *et al.* 2005, 2008; Tadros *et al.* 2007; Jeske *et al.* 2011; Pinder and Smibert 2013; Chen *et al.* 2014; Götze *et al.* 2017). In embryos from *smg* mutant females repression and degradation of a large proportion of maternal mRNAs fails (Tadros *et al.* 2007; Chen *et al.* 2014) as does zygotic production of both mRNAs and microRNAs (Benoit *et al.* 2009; Luo *et al.* 2016). In addition, the first developmental process that depends on ZGA, namely blastoderm cellularization, also fails (Dahanukar *et al.* 1999; Benoit *et al.* 2009). These results led to the conclusion that SMG function is essential for progression through the MZT (Benoit *et al.* 2009). Expression of SMG and several of its post-transcriptional co-repressors—Cup, Trailer hitch (TRAL), and Maternal expression at 31B (ME31B)—is tightly regulated at both the mRNA and protein levels, temporally restricting their function to specific phases of the MZT (Wang *et al.* 2017; Hara *et al.* 2018; Cao *et al.* 2020; Zavortink *et al.* 2020).

The highly conserved ubiquitin proteasome system (UPS) is the major pathway for targeted degradation of specific proteins across eukaryotes (Ravid and Hochstrasser 2008). This pathway consists of the E1–E2–E3 ubiquitination enzyme cascade, through which ubiquitin is sequentially transferred to the E3 ubiquitin ligase, which acts as the substrate-specificity factor that binds specific target proteins for ubiquitination and degradation through the 26S proteasome. Hundreds of E3 ubiquitin ligases have been identified across eukaryotes, belonging to several conserved classes (reviewed in Morreale and Walden 2016; Zheng and Shabek 2017).

The UPS has been implicated in several aspects of the developmental transitions from meiotically arrested oocyte to embryo and from maternal to zygotic control of development in *Drosophila* (Aviles-Pagan *et al.* 2020; Cao *et al.* 2020; Zavortink *et al.* 2020), *C. elegans* (Guven-Ozkan *et al.* 2008; Kisielnicka *et al.* 2018; Spike *et al.* 2018) and mouse (Yang *et al.* 2017). Recently, we showed that the UPS is involved in the clearance of SMG and its co-factors during the MZT (Cao *et al.* 2020): Cup, TRAL and ME31B are targeted for degradation in the middle of the MZT by the C-terminal to LisH (CTLH) E3-ligase complex, while SMG is degraded near the end of the MZT by the Skp/Cullin/F-box (SCF) E3-ligase complex. The SCF complex is one of the best studied and most highly conserved E3 ubiquitin ligases, particularly known for its role in cell cycle regulation (Bai *et al.* 1996). This complex includes the scaffold protein Cul1, the ubiquitin-binding RING-domain protein Roc1/Rbx1, and the adapter protein Skp1. The F-box is an approximately 50 amino-acid-long motif that serves as a site of protein-protein interaction either in SCF E3 ubiquitin ligase complexes or in other contexts (Kipreos and Pagano 2000). In SCF E3-ligase complexes, F-box proteins function as the substrate-binding subunit; thus, the spatio-temporal regulation of F-box protein expression can provide specificity both for the specific substrate(s) that are cleared and for where and when this occurs. While at least 45 F-box proteins have been identified in *Drosophila*, only a small subset of these has known substrates or binding sites (Dui *et al.* 2012).

We have shown that, during the MZT, SMG physically interacts with core members of the SCF complex, as well as two F-box proteins, Supernumerary limbs (SLMB) and CG14317 (Cao *et al.* 2020). Deletion of the C-terminal region of SMG, which is essential for its clearance at the end of the MZT, abrogates interaction with both of these F-box proteins (Cao *et al.* 2020). SCF^SLMB^ is a homolog of the mammalian cell cycle regulator SCF^β-TrCP^ and has previously reported roles in ovary development and cell cycle regulation in *Drosophila* (Heriche *et al.* 2003; Muzzopappa and Wappner 2005). Knockdown of SLMB or core components of the SCF complex in the early embryo results in stabilization of SMG protein (Cao *et al.* 2020). However, these proteins are loaded maternally and persist well-beyond the MZT (Cao *et al.* 2020); thus, their expression *per se* cannot explain the timing of SMG clearance by the UPS.

In contrast to the other SCF components, CG14317 protein and its cognate mRNA show an extremely temporally restricted expression pattern that coincides with ZGA and the degradation of SMG at the end of the MZT (Graveley *et al.* 2011; Casas-Vila *et al.* 2017; Cao *et al.* 2020). These observations made CG14317 an ideal candidate to function as a timer for the precise temporal degradation of SMG. Here, we show that CG14317 is required for SMG protein degradation, and that its distinctive expression pattern restricts the binding of the SCF^CG14317^ and SCF^SLMB^ complexes with SMG, to the end of the MZT. We name the *CG14317* gene *bard* and its encoded protein Bard, for Bard the Bowman’s role in targeting Smaug for destruction (Tolkien 1937).

## Materials and methods

### *Drosophila* stocks and husbandry

*Drosophila melanogaster* stocks were cultivated under standard laboratory conditions at 25°C. The “wild-type” strain used for IP-MS experiments was *w^1118^.* Additional fly strains were acquired from other labs or from the Bloomington *Drosophila* Stock Center (BDSC). The *da-GAL4* driver *P{GAL4-da.G32}* (Wodarz *et al.* 1995) used for RNAi knockdown (BDSC #55851) was a gift from T. Harris. *mCherry* RNAi was used as control (gift from T. Hurd; BDSC #35785). For immunostaining experiments *w^1118^; Df(3R)BSC510/TM6C, Sb^1^ cu^1^* (BDSC #25014) was crossed with *w^1118^; Dr^Mio^/TM3, P{w[+mC]=GAL4-twi.G}2.3, P{UAS-2xEGFP}AH2.3, Sb^1^ Ser^1^* (BDSC #6663) to produce flies carrying *Df(3R)BSC51*0 and the *twi>EGFP* balancer. Details on the generation of transgenic strains for *bard* RNAi and *bard* rescue constructs are given below.

### Embryo collection

Embryos were collected on apple juice agar plates supplemented with yeast paste from cages of adult flies in 1-h intervals (2 h for immunostaining) and aged to the desired time points at 25°C. Embryos were washed off the surface of plates with PBST (1× PBS, 0.1% Tween-20) and collected through a mesh to remove excess yeast. Embryos were dechorionated with cold 4% sodium hypochlorite for 1 min, rinsed with PBST to remove bleach, and placed on ice for further processing, described below.

### Immunoprecipitation and mass spectrometry

Embryos were collected from *w^1118^* flies and aged to 0–1 h (*i.e.*, processed immediately), 1–2, 2–3, or 3–4 h. Dechorionated embryos were crushed in a minimal volume of lysis buffer (150 mM KCl, 20 mM HEPES-KOH pH 7.4, 1 mM MgCl_2_, 0.1% Triton X-100, supplemented with protease inhibitors and 1 mM DTT), cleared by centrifugation for 15 min at 4°C and 20,000 × g, and stored at −80°C. Immediately prior to immunoprecipitation (IP), protein concentration was measured by Bradford Assay (Bio-Rad), and samples were diluted to 15 mg/ml with lysis buffer. For each IP, 250 µl of diluted lysate was mixed with 350 µg/ml RNase A, 2 µl of guinea pig anti-SMG antibody (Tadros *et al.* 2007) or guinea pig normal serum as control, and 10 µl of Protein A beads (Roche). IPs were incubated for 3 h at 4°C with end-over-end rotation. Beads were washed four times with lysis buffer, twice with lysis buffer lacking Triton X-100, then transferred to new tubes and washed twice more with lysis buffer lacking Triton X-100 to remove all traces of detergent. Downstream tryptic digest, sample preparation and HPLC/MS were the same as our previously described FLAG IP-MS protocol (Cao *et al.* 2020).

### RNAi

A transgenic construct designed to express a short hairpin RNA against *CG14317/bard* was generated. A 21-nucleotide sequence mapping to *CG14317/bard* with no predicted off-target matches of up to 16 nucleotides in the *Drosophila* genome was cloned into a microRNA scaffold in the pVALIUM22 vector (Ni *et al.* 2011), and injected into *y^1^ P{y[+t7.7]=CaryIP}su(Hw)attP8 v^1^* (BDSC #34769) by Rainbow Transgenic Flies (Camarillo, CA). The shRNA sense-strand sequence was 5′-CGATCAGTTCGACAGTTGTGT-3′, and the antisense strand sequence was 5′-ACAACTGTCGAACTGATCGGT-3′. “Maternal+zygotic” knockdown was performed by crossing females expressing the *da-GAL4* ubiquitous driver with males expressing UAS-shRNA; F1 females were then crossed to males expressing UAS-shRNA, from which embryos were collected for RNAi assays. This method resulted in 28% depletion of *bard* mRNA (Figure  2A).

### Reverse transcription and qPCR

Total RNA was collected from 0 to 4 h embryos in 200 µl (approximately 10× volume) of TRI reagent (Sigma) following the manufacturer’s protocol. cDNA was synthesized from 1 µg of total RNA per sample using the Superscript IV reverse transcriptase kit (Invitrogen), and reactions were primed using random hexamers. Reactions containing single-stranded cDNA were diluted 1:20 with ultra-pure water and used for quantification by qPCR. qPCR was performed using the Sensifast SYBr PCR mix (Bioline) following the manufacturer’s protocol. Each reaction used 5 µl of diluted cDNA, primed with gene-specific primers. Quantitative real-time PCR was performed on a Bio-Rad CFX384 Real-Time System, and gene expression was analyzed using the Bio-Rad CFX manager. *bard* expression was averaged across three technical replicates and normalized to the *RpL32* control.

### Western blotting

Dechorionated embryos were counted, then crushed using a microcentrifuge tube pestle in SDS-PAGE sample buffer at a concentration of 1 embryo/µl and boiled for 2 min. Eight microliter per sample was loaded in 6% SDS-PAGE and subsequently transferred to PVDF membrane. Blots were blocked at room temperature with 2% nonfat milk in PBST (0.1% Tween-20) for 30 min, and incubated at 4°C overnight with primary antibody (1:20,000 guinea pig anti-SMG; 1:50,000 mouse anti-Tubulin [Sigma T5168]) diluted in blocking solution. Blots were washed for 3 × 10 min with PBST at room temperature with rocking, and incubated with 1:5000 HRP-conjugated secondary antibodies (Jackson ImmunoResearch) in blocking solution at room temperature for 1 h. Blots were washed again for 3 × 15 min with PBST, developed using Immobilon Luminata Crescendo Western HRP substrate (Millipore), imaged using ImageLab (Biorad), and quantified using ImageJ. Details of the antibodies used can be found in the Reagents Table.

### Transgenic rescue constructs

An approximately 7.8 kb genomic fragment containing *CG14317/bard*, and extending 3.9 kb upstream and 2.3 kb downstream of the transcript (diagrammed in Figure  3A), was PCR amplified from *w^1118^* genomic DNA using primers flanked with *Bam*HI and *Not*I restriction sites. This endogenous rescue construct was inserted by restriction digest and ligation into the pCaSpeR4 cloning vector containing an attB site (Tadros *et al.* 2007; Markstein *et al.* 2008). For the 3xFLAG-tagged rescue construct, the PCR-amplified genomic fragment was first ligated into the pUC19 backbone, and an N-terminal 3xFLAG coding sequence was inserted after the start codon by site-directed mutagenesis. The 3xFLAG-tagged rescue fragment was excised with *Bam*HI and *Not*I, and cloned into pCaSpeR4 as described above. Rescue fragments in pCaSpeR4 were integrated into an attP40 landing site on the second chromosome (Markstein *et al.* 2008) using the phiC31 integrase method by Rainbow Transgenic Flies (Camarillo, CA), and crossed to the third chromosome deficiency for rescue experiments.

### Immunostaining

Two- to four-hour old embryos were collected and dechorionated as described above. Dechorionated embryos were fixed and permeabilized in 4% formaldehyde and heptane for 40 min, and devitellinized by addition of methanol followed by vigorous shaking for 30 s. Fixed embryos were rehydrated by washing 4 times with PBSTx (1xPBS + 0.1% Triton X-100) and blocked with 10% bovine serum albumin (BSA) in PBSTx for 1 h at room temperature. Embryos were incubated at 4°C overnight with primary antibodies (1:1000 guinea pig anti-SMG and 1:200 mouse anti-GFP [Sigma #G6539]) diluted in 1% BSA in PBSTx, with rocking. Embryos were washed 3 × 15 min with PBSTx at room temperature, then incubated in secondary antibodies (1:300 Cy3-conjugated donkey anti-guinea pig [Jackson ImmunoResearch], 1:300 goat anti-mouse Alexa Fluor 488 [ThermoFisher]) for 1 h rocking at room temperature. Embryos were washed 5 × 10 min with PBSTx, and mounted in 2.5% DABCO, 70% glycerol in PBS. Details of antibodies used can be found in the Reagents Table. Images were collected using a Zeiss AxioSkop-2 MOT fluorescence microscope and the QCapture Suite PLUS acquisition software. Average fluorescence intensity of each embryo was quantified in ImageJ.

### Statistical analysis

The ProHits software package (Liu *et al.* 2010) was used to perform peptide validation and protein interaction analysis. Proteins with associated peptide counts were filtered for iProphet probability >0.95 and number of unique peptides ≥2 (Shteynberg *et al.* 2011). Significance Analysis of INTeractome (SAINT) was used to determine the probability of each interacting protein (Choi *et al.* 2011). SAINTexpress was run on the ProHits interface with nburn = 2000; niter = 5000; lowMode = 1; minFold = 1; normalize = 1; no compression for bait (*n* = 3) or control (*n* = 2). Dot plot of the SAINT analysis was generated through ProHits-viz (Knight *et al.* 2017). Detailed results of the SAINT analysis for all interactions are listed in Supplementary Table S1.

All other graphing and statistical analyses were carried out using GraphPad PRISM 8.3. Quantification of Western blots comparing SMG expression in control *vs. bard* RNAi across 3 biological replicates were analyzed using the two-tailed Student’s *t*-test. Quantification of IF, comparing SMG expression in GFP(+) *vs* GFP(−) embryos were performed for at least 10 embryos per category, and analyzed using the two-tailed Mann–Whitney *U*-test.

## Results

### Bard is expressed and interacts with Smaug at the end of the MZT

**Figure 1:**
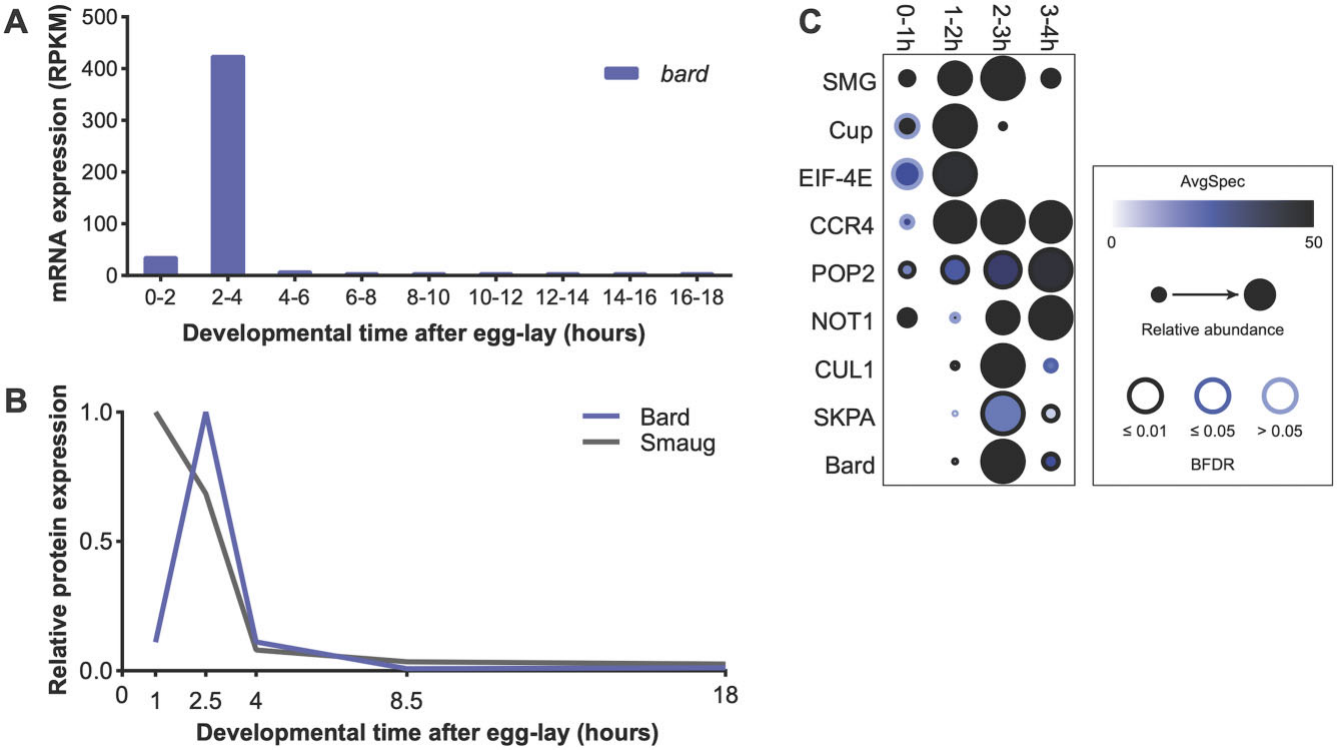
*bard* mRNA and Bard protein are expressed in a narrow time window, coinciding with SCF^Bard^ binding to SMG and its clearance from the embryo at the end of the MZT. (A) Developmental transcriptome (Graveley *et al.* 2011) captures *bard* transcript expression almost exclusively at 2–4 h after egg-lay. (B) A developmental proteomic study (Cao *et al.* 2020) shows that Bard protein expression closely correlates with that of its cognate transcript and is coincident with SMG protein clearance. (C) Dot-plot of developmental IP-MS experiments of SMG over the first 4 h of embryo development for interactions with its co-regulators and the SCF complex. Average spectral counts, relative abundance normalized to control IP, and BFDR of each significant interaction based on SAINT analysis are plotted. Raw data are in Supplementary Table S1. Note that SMG peptide abundance relative to control IP captures over these timepoints, closely resembles total SMG levels in the embryo.

To compare the expression of Bard to that of SMG we used previously published transcriptomic and proteomic datasets. From modENCODE transcriptome data (Graveley *et al.* 2011), *bard* mRNA is present at very low levels in embryos 0–2 h after egg-lay (38 RPKM), accumulates to very high levels by 2–4 h (427 RPKM), and decreases more than 40-fold by 4–6 h (to 10 RPKM; Figure  1A). The *bard* transcript is not detected at any other stage during embryogenesis and is absent from ovaries. Thus, *bard* is a strictly zygotic mRNA that is synthesized in a burst near the end of the MZT and then is cleared from the embryo.

Next, we plotted the expression pattern of the Bard and SMG proteins in the early embryo using our previously published embryo proteome study (Cao *et al.* 2020). The Bard protein expression pattern is almost identical to that of its cognate mRNA: Bard is absent from the early embryo, accumulates rapidly toward the end of the MZT coincident with degradation of SMG, and is rapidly cleared from the embryo shortly thereafter (Figure  1B). An independent proteomic study is consistent with this Bard expression profile (Casas-Vila *et al.* 2017).

Given the temporal correlation between Bard accumulation and SMG protein degradation, we asked whether Bard and the other components of the SCF E3 ligase complex bind SMG specifically during this time window. To investigate the temporal interaction between SMG and SCF^Bard^, we performed a time-course immunoprecipitation followed by mass spectrometry (IP-MS) on endogenous SMG over the first 4 h of embryo development. As a control for the time-course we examined SMG’s interactions with its translational co-repressor, Cup, which is an eIF-4E-binding protein, as well as with eIF-4E itself. Cup is known to be cleared by the middle of the MZT (Cao *et al.* 2020) and, as expected, in our time-course, interactions were largely lost by 2–3 h (Figure  1C). As a second control, we looked at SMG’s interactions with the deadenylase-complex components CCR4 (Twin), POP2 and NOT1, which SMG recruits to destabilize its targets and which are expressed throughout the MZT (Semotok *et al.* 2005; Cao *et al.* 2020). These proteins showed their strongest interactions with SMG after 0–1 h, and continuing throughout the rest of the time-course (Figure  1C). These results agree with previous studies suggesting that SMG continues to function to direct maternal RNA destabilization after the degradation of its translational co-repressors, and further support the hypothesis that there is a “repression-to-degradation switch” in transcript regulation during the MZT (Wang *et al.* 2017; Cao *et al.* 2020). We note that the SMG protein we immunoprecipitated at the 3–4 h time-point is likely to derive in large part from the primordial germ cells (PGCs), where SMG protein persists at high levels and continues to function in mRNA degradation (Siddiqui *et al.* 2012).

Confident that our time-course was accurate, we then assessed the interaction of SMG with the SCF^Bard^ subunits CUL1, SKPA, and Bard. As expected, Bard almost exclusively interacted with SMG at 2–3 h (Figure  1C), during the time when Bard accumulates in the embryo and SMG is being degraded. Strikingly, CUL1 and SKPA interaction with SMG was almost identical to that of Bard despite the fact that these proteins are maternally loaded and present at constant levels throughout the MZT (Cao *et al.* 2020). These results are consistent with the hypothesis that expression of the substrate-recognition F-box subunit, Bard, is required for binding of the SCF complex to SMG at the end of the MZT.

### Bard is required for degradation of SMG at the end of the MZT

**Figure 2:**
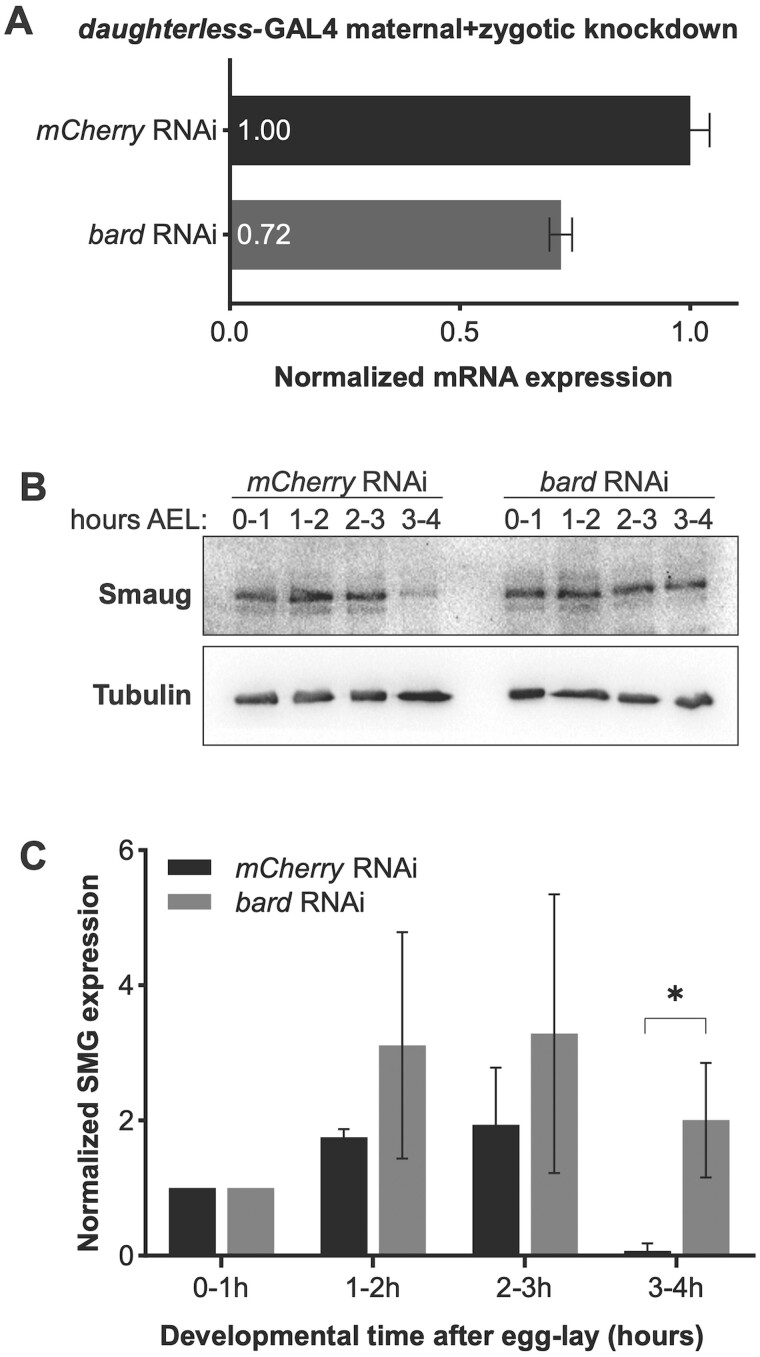
RNAi knockdown of *bard* in the early embryo results in stabilization of SMG protein. (A) Maternal + zygotic knockdown of *bard* transcripts. Zero- to four-hour old embryos were collected from flies expressing *UAS-shRNA* directed against *bard* under control of the *daughterless-GAL4* driver (see *Materials and Methods*). There was 28% depletion of *bard* mRNA relative to control knockdown with *mCherry* shRNA. Note: Protein expression could not be assayed due to lack of an antibody against Bard. (B) Western blot of embryos collected over the first 4 h after egg-lay (AEL) from *mCherry* control knockdown or *bard* knockdown. The *mCherry* control is almost identical to the previously reported time course for wild type (*cf*. Figure  4, lanes 10–13 in Benoit *et al.* 2009). SMG persists at high levels 3–4 h AEL when *bard* is knocked down in the embryo by RNAi. (C) Quantification of SMG expression, normalized to the α-Tubulin loading control, across three biological replicates. *P < 0.05, Student’s *t*-test.

To determine whether Bard is required for the degradation of SMG at the end of the MZT, we first knocked down *bard* transcripts in the early embryo using RNAi and examined the effect on the temporal profile of SMG protein. To do so, we cloned an shRNA targeting *bard* mRNA into a UAS expression vector optimized for germline expression (see *Materials and Methods*). Transgenic females carrying both the UAS-*bard shRNA* transgene and the ubiquitous *daughterless-GAL4* driver express shRNA against *bard* during oogenesis, thus their embryos have maternally supplied shRNA. By crossing these females to males expressing *UAS-bard* shRNA we maximized knockdown via “maternal + zygotic” (M + Z) RNAi (Figure  2A; 28% knockdown was achieved). Despite the limited knockdown, we found that knockdown of *bard* in the embryo resulted in significant stabilization of SMG protein (Figure  2, B and C). In the *mCherry* control knockdown, SMG was largely depleted by 3–4 h after egg-lay, as expected. In contrast, in *bard* M + Z shRNA knockdown embryos SMG persisted at this time point, suggesting that depletion of Bard leads to stabilization of SMG protein.

**Figure 3:**
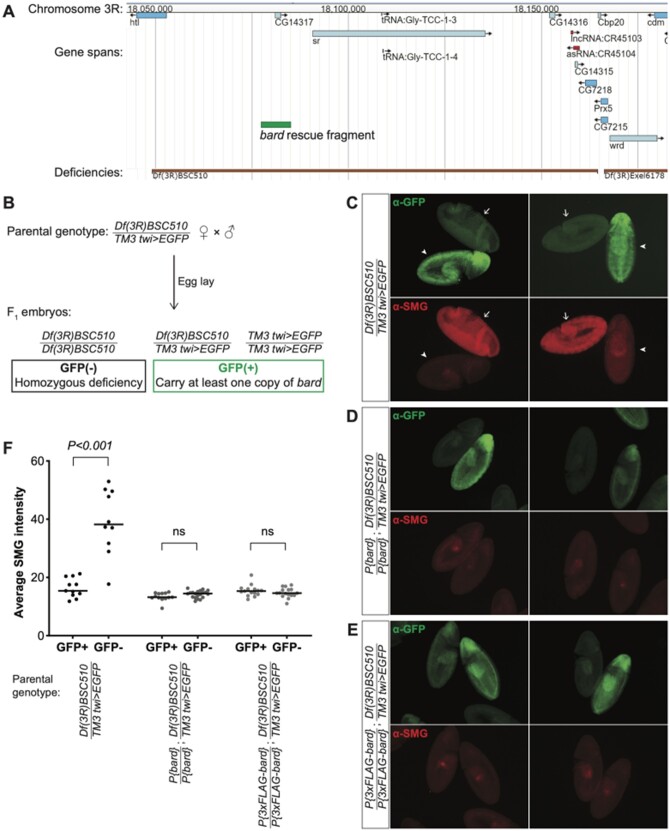
Bard is required for the degradation of SMG in the embryo at the end of the MZT. (A) JBrowse genome browser snapshot of the region deleted by *Df(3R)BSC510*, which includes *bard* (*CG14317*) and several neighboring genes on Chromosome 3R. The *bard* rescue fragment used in panels D and E is annotated in green. (B) Embryos were collected from flies carrying *Df(3R)BSC510* over a *twist (twi)>EGFP* balancer as described in *Materials and Methods*. Homozygous deficiency embryos could be distinguished from embryos carrying the balancer by lack of GFP staining. (C) Among embryos undergoing gastrulation, those carrying the balancer chromosome show low levels of SMG expression (GFP+, arrowheads) comparable to clearance of SMG in wild-type, whereas homozygous deficiency embryos show significantly higher, ubiquitous SMG expression (GFP−, arrows). This post-MZT persistence of SMG expression is rescued with either: (D) a transgene expressing Bard, or (E) a transgene expressing 3×FLAG-tagged Bard. (F) Quantification of SMG intensities of embryos with the indicated genotypes, of which examples are shown in panels B–D; *n* > 10 for each group, ns: not significant, Mann–Whitney *U*-test.

To validate the role of Bard in SMG protein degradation, we made use of a chromosomal deficiency that deletes approximately 115 kb from chromosome 3R, including the *bard* locus (Figure  3A). *Df(3R)BSC510* deletes *CG14317/bard*, in addition to *CG14316, CG7218, CG14315, CG14316, heartless, stripe;* the noncoding RNAs *CR45104* and *CR45103*; and two tRNAs (Cook *et al.* 2012). By generating a stock carrying this deficiency over a *twist>EGFP* balancer, we could easily distinguish by the absence of GFP, those embryos that were homozygous for *Df(3R)BSC510*, thus lacking *bard*, from GFP-positive embryos that carried the balancer chromosome (Figure  3B). Other than *bard*, most of the genes deleted by this deficiency are not expressed in the embryo during the time period we were investigating. Exceptions are: the *heartless* transcript, which is moderately expressed in the early embryo, but Heartless protein is known to be expressed and function at a later stage in embryo development (Beiman *et al.* 1996); and *CG7218*, a maternally loaded mRNA with no known role in embryogenesis. Thus, we reasoned that the deficiency was unlikely to affect early embryo development. In agreement with this, we observed that the GFP(−) homozygous deficiency embryos developed normally through the MZT and early gastrulation, comparable to their GFP(+) siblings (Figure  3, C–E).

Strikingly, while SMG protein in embryos heterozygous for *Df(3R)BSC510* or homozygous for the balancer chromosome was depleted from the bulk cytoplasm by cellularization (*i.e.*, prior to gastrulation), homozygous deficiency embryos showed ubiquitous and high SMG expression that persisted well beyond the onset of gastrulation (Figure  3, C and F). These results provide further evidence that Bard is required for degradation of SMG at the end of the MZT. We note that it was not possible in this experiment to distinguish *Df(3R)BSC510/Balancer* from *Balancer/Balancer* embryos since both genotypes were GFP(+) (Figure  3B). Thus, we were unable to assess whether there was partial stabilization of SMG in the deficiency heterozygotes as might be predicted from the knockdown experiment. We also note that the stage assayed in this experiment (germband extended) is later than that in the knockdown so the data are not directly comparable.

Finally, to confirm Bard’s role in SMG clearance, we produced two independent transgenic lines carrying, inserted on the second chromosome, a small genomic region that includes *bard* but none of the other genes deleted by *Df(3R)BSC510* (Figure  3A). Embryos expressing either Bard or an N-terminal FLAG-tagged version of Bard under control of its endogenous regulatory elements, were sufficient to fully rescue the defect in SMG degradation observed in homozygous deficiency embryos (Figure  3, D–F). Taken together, these data provide strong genetic evidence that Bard targets SMG for clearance at the end of the *Drosophila* MZT.

## Discussion

Bard was predicted to be an SCF component based on presence of an F-box domain (Dui *et al.* 2012). However, it had no known substrate and its function as SCF E3 ligase complex subunit in *Drosophila* had not been established. Previously, we noted that the combination of Bard’s distinctive expression profile in the early embryo and its physical interaction with SMG supported a potential role as an SCF component that might confer precise temporal regulation on clearance of SMG at the end of the MZT (Cao *et al.* 2020). Here we have presented proteomic data that support a role for Bard as a “timer” for recruitment of SCF to SMG as well as genetic evidence that Bard is required for the degradation of SMG at the end of the MZT. An earlier study found that SMG is not degraded in activated, unfertilized eggs, which undergo maternally directed post-transcriptional processes (*e.g.*, mRNA destabilization) but do not undergo transcriptional activation of their genome (Bashirullah *et al.* 1999; Benoit *et al.* 2009), leading to the hypothesis that zygotic transcription is required for SMG clearance. Since *bard* mRNA and Bard protein are exclusively zygotically synthesized (Figure  1, A and B), our data provide a mechanistic basis for the dependence of SMG degradation on activation of zygotic transcription.

**Figure 4:**
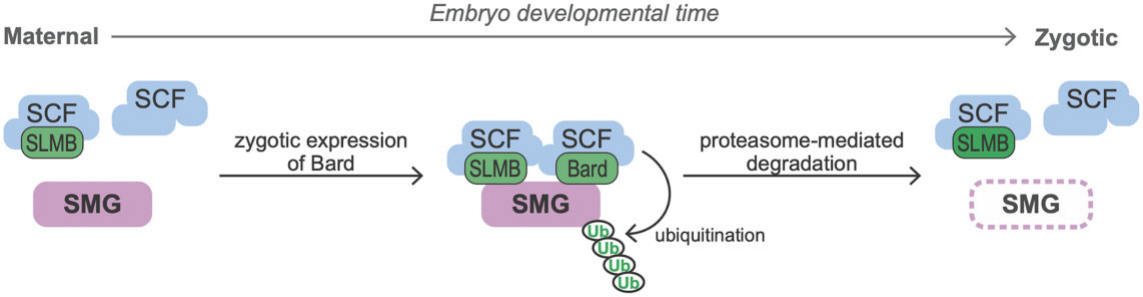
Model of the temporal regulation of SMG protein degradation by SCF^Bard^ during the MZT. During the maternal phase of the MZT, although the SCF core components and the F-box protein SLMB are present they do not bind SMG. Zygotic synthesis of Bard toward the end of the MZT promotes binding to SMG of SCF^Bard^ and SCF^SLMB^. This triggers ubiquitin-mediated proteasomal degradation of SMG.

There is now evidence that two F-box proteins—SLMB (Cao *et al.* 2020) and Bard (this study)—are required for degradation of SMG at the end of the MZT. Although SLMB as well as the core components of the SCF complex (CUL1, SKPA, and ROC1a) are all expressed at relatively constant levels in the early embryo, here, we have shown that the SCF complex does not interact with SMG until the end of the MZT, coinciding with accumulation of Bard in the embryo. The mammalian homolog of SLMB, the cell cycle regulator β-TrCP, has been shown to function in heterodimeric complexes with another F-box protein to increase the efficiency of the ubiquitination of its target protein (Tang *et al.* 2007). Thus, it is entirely possible here that SLMB and Bard function cooperatively to recruit the SCF complex to SMG for ubiquitination. Such a model highlights Bard’s temporally restricted expression in the embryo as a timer for the action on SMG of the SCF E3-ligase complex as a whole (Figure  4). We note that, while SLMB was found to interact with SMG in our previous IP-MS experiments (Cao *et al.* 2020), it did not pass the threshold as a significant interactor of SMG in our time-course IP-MS at any of the time points, possibly because of the small scale of these experiments due to the technical challenge of collecting sufficient material in narrow time windows. Future IP-Western blot or IP-MS experiments may provide additional insights into the cooperative binding model by asking, for example, whether Bard interacts with SMG in the absence of SLMB and *vice versa*. Such experiments would require appropriate mutations in both genes; currently no mutations are available in the *bard* gene.

Multiple mechanisms restrict SMG expression to the MZT. Although *smg* mRNA is expressed during oogenesis, it is kept translationally repressed until the Pan gu kinase complex relieves repression upon egg activation (Tadros *et al.* 2007). Misexpression of SMG in in the germline of ovaries results in failure to progress through the stages of oogenesis (Semotok *et al.* 2005). SMG also needs to be cleared at the end of the MZT. Persistence of SMG beyond the MZT leads to a reduction in the levels of zygotically expressed transcripts carrying *cis*-elements that can be bound by SMG (Cao *et al.* 2020). By timing the clearance of SMG, Bard thus plays a crucial role in the orderly handover from maternal to zygotic control of the transcriptome.

SMG is not the only protein that is cleared at the end of the MZT: Our previous proteomic analyses (Cao *et al.* 2020) identified additional proteins that are known to have important roles in the early embryo and show a similar degradation profile to SMG, including the germ plasm RNA-binding proteins Vasa, Oskar, and Tudor. During the MZT, *Bard* is transcribed in the soma but not the PGCs (see http://flyexpress.net/search/genes/CG14317/images/BDGP/LDVO) (Konikoff *et al.* 2012). This is consistent with the fact that the PGCs are transcriptionally silent after they bud from the posterior of the early embryo (Van Doren *et al.* 1998; Hanyu-Nakamura *et al.* 2008); budding occurs prior to Bard synthesis in the bulk cytoplasm. We showed previously that SMG persists in PGCs after it is cleared from the bulk cytoplasm (Siddiqui *et al.* 2012). We speculate that the presence of SMG, Vasa, Oskar, and Tudor in the PGCs may, at least in part, be due to the absence of Bard.

Additional maternal proteins with a similar decay profile to SMG include the histone H1 variant BigH1 and the cell cycle regulatory components APC7, Cyclin B, and Scrambled, among others (Cao *et al.* 2020). Intriguingly, like SMG, both Scrambled and BigH1 have specific roles that are restricted to the MZT. Scrambled is required for actin organization during syncytial divisions in the early embryo but is not required for blastoderm cellularization or post-cellularization mitotic divisions (Stevenson *et al.* 2001). BigH1 keeps the zygotic genome silent and aids in rapid nuclear divisions in the early embryo, while clearance of BigH1 from the chromatin and replacement with histone H1 at the end of the MZT is required to permit ZGA (Pérez-Montero *et al.* 2013; Henn *et al.* 2020). Thus, like SMG, precisely timed degradation of these proteins at the end of the MZT is important for an orderly developmental progression. Future experiments will reveal whether these proteins are also substrates of SCF^Bard^.

## Data availability

New fly strains and plasmids generated in this article are available upon request. Source data plotted in Figure  1A were extracted from FlyBase (flybase.org). Source data plotted in Figure  1B were extracted from Supplementary Table S1 of our previous study (Cao *et al.* 2020). The full list of significant interactors from SAINT analysis of time-course IP-MS experiments presented in Figure  1C is provided in Supplementary Table S1. Raw mass spectrometry data used for these analyses have been deposited to ProteomeXchange (MassIVE MSV000088199, PXD028973) (ftp://massive.ucsd.edu/MSV000088199/).

Supplementary material is available at *GENETICS* online.

## Supplementary Material

iyab177_Supplementary_Data
