# Supplementary material for: The F-box protein Bard (CG14317) targets the Smaug RNA-binding protein for destruction during the Drosophila maternal-to-zygotic transition
Source: Genetics. 2021 Oct 20;220(1):iyab177. doi: 10.1093/genetics/iyab177 (PMC8733446; doi:10.1093/genetics/iyab177)
Supplement: iyab177_Supplementary_Data [file iyab177_supplementary_data.zip › iyab177-suppl_data/GENETICS-GENETICS-2021-304739-s03.docx]

**SUPPLEMENTAL MATERIALS LEGEND**

Table S1: SAINT analysis of the time-course SMG IP-MS results

Reagent table
